# Impact of pre- and post-variant filtration strategies on imputation

**DOI:** 10.1038/s41598-021-85333-z

**Published:** 2021-03-18

**Authors:** Céline Charon, Rodrigue Allodji, Vincent Meyer, Jean-François Deleuze

**Affiliations:** 1 CEA Paris-Saclay, Institut François Jacob, Centre National de Recherche en Génomique Humaine, 2 rue Gaston Crémieux, Evry, 91057 France; 2 Radiation Epidemiology Group CESP, Inserm Unit 1018, Gustave Roussy Université Paris Saclay, 114 rue Edouard Vaillant, Villejuif, 94805 France

**Keywords:** Quality control, Genetic markers, Genotype

## Abstract

Quality control (QC) methods for genome-wide association studies and fine mapping are commonly used for imputation, however they result in loss of many single nucleotide polymorphisms (SNPs). To investigate the consequences of filtration on imputation, we studied the direct effects on the number of markers, their allele frequencies, imputation quality scores and post-filtration events. We pre-phrased 1031 genotyped individuals from diverse ethnicities and compared the imputed variants to 1089 NCBI recorded individuals for additional validation. Without QC-based variant pre-filtration, we observed no impairment in the imputation of SNPs that failed QC whereas with pre-filtration there was an overall loss of information. Significant differences between frequencies with and without pre-filtration were found only in the range of very rare (5E−04–1E−03) and rare variants (1E−03–5E−03) (p < 1E−04). Increasing the post-filtration imputation quality score from 0.3 to 0.8 reduced the number of single nucleotide variants (SNVs) < 0.001 2.5 fold with or without QC pre-filtration and halved the number of very rare variants (5E−04). Thus, to maintain confidence and enough SNVs, we propose here a two-step filtering procedure which allows less stringent filtering prior to imputation and post-imputation in order to increase the number of very rare and rare variants compared to conservative filtration methods.

## Introduction

First generation genome-wide association studies (GWAS) characterised many common single nucleotide polymorphisms (SNPs) seldom considered as the main cause of disease even at genome-wide significance^1–3^. Despite implementation of genomics imputation to improve the statistical power of association analyses^4,5^, SNPs were identified with small effect sizes on disease risk^6^.

Initial filtration of single nucleotide variants (SNVs) (pre-filtration) was considered necessary to warrant correct inference of SNPs during imputation^7,8^. This was mostly based on routine quality control (QC) applied in association studies and fine mapping. The QC excluded low frequency variants and singletons^9,10^. The confidence index threshold for post-imputation information measures was set either between 0.3 and 0.4 or at a more conservative score of 0.7–0.9^6,11,12^.

Imputation increased the number of SNPs for non-genotyped variants in individuals, leading to higher power to detect significant associations^4,13,14^. Common imputation methods based on the graphical model of a haplotype set applied in Beagle^15–19^ and the Hidden Markov Model (HMM) implemented in IMPUTE^20^ and MaCH^21^ showed comparable accuracy^16,22^. Early GWAS imputation analyses used reference panels of the International HapMap project ^23–25^. The reference genomes were subsequently improved to characterise low and rare variants by sequencing larger sets of individuals from the 1000 Genome project phase 1 (1000GP1)^26^ and phase 3 (1000GP3)^27^ and from other panels of the haplotype reference consortium (HRC)^28^, the UK10K^29^ and the NHLBI Trans-Omics for precision Medicine (TOPMed)^30^. Structural variants were further uncovered with the 1000GP3^31^ and the genome of the Netherlands project (GoNL)^32^. The imputation of rare variants was improved after rephasing the reference sequence of the 1,092 genomes (1000GP1) with SHAPEIT2 and Beagle against the 1000GP3^33,34^.

The interest in imputation of rare variants in disease gene discovery was first illustrated by Jonsson et al. for Alzheimer’s disease^35,36^. The effects of rare variants in diseases are currently being analysed in both GWAS and fine-scale mapping association studies^37^. Rare variants are difficult to investigate; in many of these studies, the SNPs of individuals are routinely removed prior to imputation^7,8^, which can lead to a loss of information or loss of accuracy when imputing the unaccounted for SNPs that may be in linkage disequilibrium (LD) with SNVs^38^. The effect of QC prior to imputation is not fully understood in relation to downstream processes^39^. Understanding the underlying effects of filtration on imputation in the 1000GP should provide insight that could be applied to the larger reference panels. It has been shown that filtering out low quality SNVs rather than incorporating them with a low quality score weight^40,41^, can decrease the power of locus-based approaches when the causal variant is of good quality. A quality control that is too stringent can remove many variants, therefore to avoid variant exclusion, less stringent quality control or no pre-filtration may be required^3,42^. Das et al.^43^ also found that SHAPEIT performed better with less missing variants. Furthermore, missing genotypes can have an impact on quality assessment based on the certainty and the agreement of true genotypes^3^.

We performed an imputation without SNP pre-filtering to see if it would generate rare and more common SNPs of reliable confidence and accuracy. We also examined the imputation results after downstream filtration under stringent and non-stringent conditions. We used the re-phased 1000GP which satisfies criteria for reliable imputation accuracy of common and low frequency variants^34,36,44^ for our sample set of 1031 individuals which are similar in terms of size and population origin^16,45,46^.

Based on our findings, we recommend calculating the MAF of the sample set and suggest using it during pre-filtration to minimise the loss of rare variants potentially important for disease risk discovery, and to improve imputation quality. We also propose new approaches for post-imputation filtration with a 2-step strategy using (1) the MAF and (2) the quality score.

## Methods

We used empirical sample datasets for 1,031 individuals from a 2 Mb region of chromosome 20 file source: https://mathgen.stats.ox.ac.uk/genetics_software/shapeit/shapeit.html.

A full description of the samples (including 37% AMR, 34.3% EUR, 25.1% EAS, 2.95% AFR and 0.65% SAS) is available at: ftp://ftp.1000genomes.ebi.ac.uk/vol1/ftp/technical/working/20130606_sample_info/20130606_sample_info.xlsx. We pre-phased sample genotypes prior to imputation with SHAPEIT v2.r790.RHELS_5.4 for former ordering of the markers to provide better downstream accuracy and increase imputation speed^47^. SHAPEIT2^45^ was run, followed by imputation with IMPUTE2^14^ against a known reference haplotype^32^ based on the 1,092 individuals from phase 1 (35% EUR, 26% ASN, 22% AFR, 17% AMR) of the 1000GP, release 2011–05-21: ftp://ftp.1000genomes.ebi.ac.uk/vol1/ftp/phase1/analysis_results/shapeit2_phased_haplotypes/.

The same reference was previously re-phased with the coordinates of the NCBI build 37 (hg19)^25^ and made available in 2015: https://mathgen.stats.ox.ac.uk/impute/data_download_1000G_phase1_integrated_SHAPEIT2_16-06-14.html.

After imputation, the MAF was determined for all variants and compared with that of the NCBI gMAF dbSNPB137 which is based on 1089 individuals from the 1000GP1.

We clustered the variants after imputation in different levels of non-overlapping MAFs, such as null (0–1E−04], very rare (1E−04–1E−03], rare (1E−03–1E−02], low (1E−02–5E−02], common (5E−02–1E−01] and high (1E−01–5E−01] frequencies. The very rare and rare classes were sub-divided into 2 bins, (1E−04–5E−04] and (5E−04–1E−03] for very rare MAFs (1E−03–5E−03] and (5E−03–1E−02] for the rare MAFs.

The minimum imputed MAF inferred from the allele dosage, based on genotype probabilities generated by IMPUTE2, was 1E−04 which corresponds to less than one heterozygous-imputed genotype. In our sample set and dbSNPB137, the minimum MAF was 5E−04, referring to at least one heterozygote genotyped individual, thus demonstrating greater reliability compared with the dosage probabilities. NCBI uses the global 1000GP data to determine the minor alleles (gmaf) and frequencies^18^.

In order to include the conditions without filtration and avoid missingness, all variants and genotyped individuals were primarily maintained for downstream analysis**.** After quality control (QC) 17.5% of variants were removed (Supplementary materials). The individuals were all retained as their pass scores for QC conditions were in the range used by most GWAS. To maintain similar conditions using SHAPEIT2 and IMPUTE2, the same seed (i.e. 1411139299) was used in each case before and after QC. The difference between imputation runs was therefore based on the number of variants, which served as the main variable for monitoring their effects.

To verify whether 2 Mb was representative of all regions in the genome, each chromosome from the 1000GP1 (included in the dbSNPB137) was split every 2 Mb. We compared the SNP counts, density and MAF of the regions with the 1000GP1 region studied (Supplementary Fig. S1, S2, Table S1).

Statistics were performed with the R package, version R 3.2.4^48^. The Wilcoxon paired test was performed for each class of frequencies to compare the MAF and information-impute2 scores^14,45^ between imputation runs in the presence or absence of QC. The Wilcoxon unpaired test was done for each class to compare the MAF of genotypes or imputed variants with that of dbSNP. Tests performed were two-sided unless specifically indicated as one-sided.

## Results

### Genotype imputation conditions

We focused on an arbitrarily chosen locus representing the size of a fine-scale mapping region that lies within Chr20, commonly used as a standard chromosome^49,50^. This allowed us to compare both imputation runs in the presence or absence of QC using the same seed for reproducibility.

Before imputation, the main cause for removal of rare variants during pre-filtration of the genotyped SNPs was the MAF selection criterion of 0.01, i.e. the standard used in most GWAS and fine-scale mapping studies (Supplementary materials). The number of genotyped SNPs dropped from 1762 to 1452 after pre-filtration.

The number (27,090) and density of SNPs to impute (13.545/Kb) for the 2 Mb region studied were within the standard deviation (sd) (Table S1) and the interquartile range (IQR) of the average counts and density of SNPs for each 2 Mb of the genome (Supplementary Fig.S1). The MAF was also within the IQRs of the MAF on the Chr20 and of the whole genome for every 2 Mb (Supplementary Fig.S2). Omitting common filtration did not impact the number of imputed variants and it conserved the initial density of SNPs in the region.

### Genotype accuracy

To increase imputation accuracy we used the re-phased haplotypes from the 1000GP^25,45^*.* The overall concordance^34^ between original and imputed genotypes was 98.83%. We then looked at the square correlation defined in IMPUTE2 (r2_type0), a more stringent coefficient that determines the accuracy of each imputation compared with the masked genotyped SNPs^27,34^. The threshold for r2_type0 outliers obtained for the imputed genotypes was < 0.825, of which just 44% had a genotyped MAF < 0.01. Therefore, the SNP frequency < 0.01 (the criterion generally used to remove variants prior to imputation) was not the main cause for failing this metric. For these outliers, the more commonly used information-impute2 metric had a maximum value of 1.

The majority of imputed variants (68%) had a MAF of < 0.01 (Table 1). Thus downstream imputation parameters were mostly not affected by the MAF when variants with a MAF < 0.01 before imputation were retained. Additionally, the imputation accuracy of the genotype variants (r2_type0) without pre-filtration compared to those with pre-filtration was significantly higher for the high category of MAFs (p-value = 1E−06, one-tailed).

**Table 1:** Variants counts and Wilcoxon paired signed rank test of minor allele frequencies before and after QC.

maf categories	no QC	QC	maf noQC		maf QC		Wilcoxon paired test
	n	n	Mean	sd	Mean	sd	maf two-sided p-value
0-0	3292	3299	0.00	0.00	0.00	0.00	< 2.20 E-16 ****
1E−04-5E−04	3778	3827	3.00 E−04	1.30 E−04	2.90 E−03	1.30 E−04	5.97 E−01
5E−04-1E−03	3139	3103	7.30 E−04	1.60 E−04	7.30 E−04	1.60 E−04	1.49 E-11 ****
1E−03-5E−03	6574	6518	2.4 E−03	1.10 E−03	2.40 E−03	1.10 E−03	8.99 E-11 ****
5E−03-1E−02	2119	2127	7.1 E -03	1.40 E−03	7.00 E−03	1.40 E−03	2.14 E−01
1E−02-5E−02	3630	3668	2.3 E−02	1.10 E−02	2.29 E−02	1.11 E−02	4.55 E−01
5E−02-1E−01	955	967	7.3 E−02	1.40 E−02	7.30 E−02	1.40 E−02	9.20 E−01
1E−01-5E−01	4316	4287	2.80 E−01	1.20 E−01	2.80 E−01	1.20 E−01	7.91 E−01
0-5E−01	27803	27796	5.00 E−02	1.10 E−01	5.00 E−02	1.10 E−01	5.12 E−01

No significant differences were found in the total number (n) of variants before (no QC) and after QC (QC) with the Wilcoxon paired test (p value = 9.45 E−01) When stratifying each variant in the subclasses, significant differences in the maf were observed for the null alleles and one subclass of very rare (5E−04-1E−03) and rare variants (1E−03-5E−03).

Levels of significance **** < 0.0001, *** < 0.001, ** < 0.01, * < 0.05.

Percentage of SNPs with null MAF 12%, very rare MAF 24.8%, rare MAF 31.1%, low MAF 13.1%, common MAF 3.5%, SNP high MAF 15.5%.

Percentage of rare variants < 0.01 is higher (68% including the null alleles and 56% without the null alleles) compared to the SNP MAF > 0.01 (32%).

*maf* minor allele frequencies, *n* number of variants, *sd* standard deviation, *QC* quality control.

### MAF comparisons between runs

**Figure 1:**
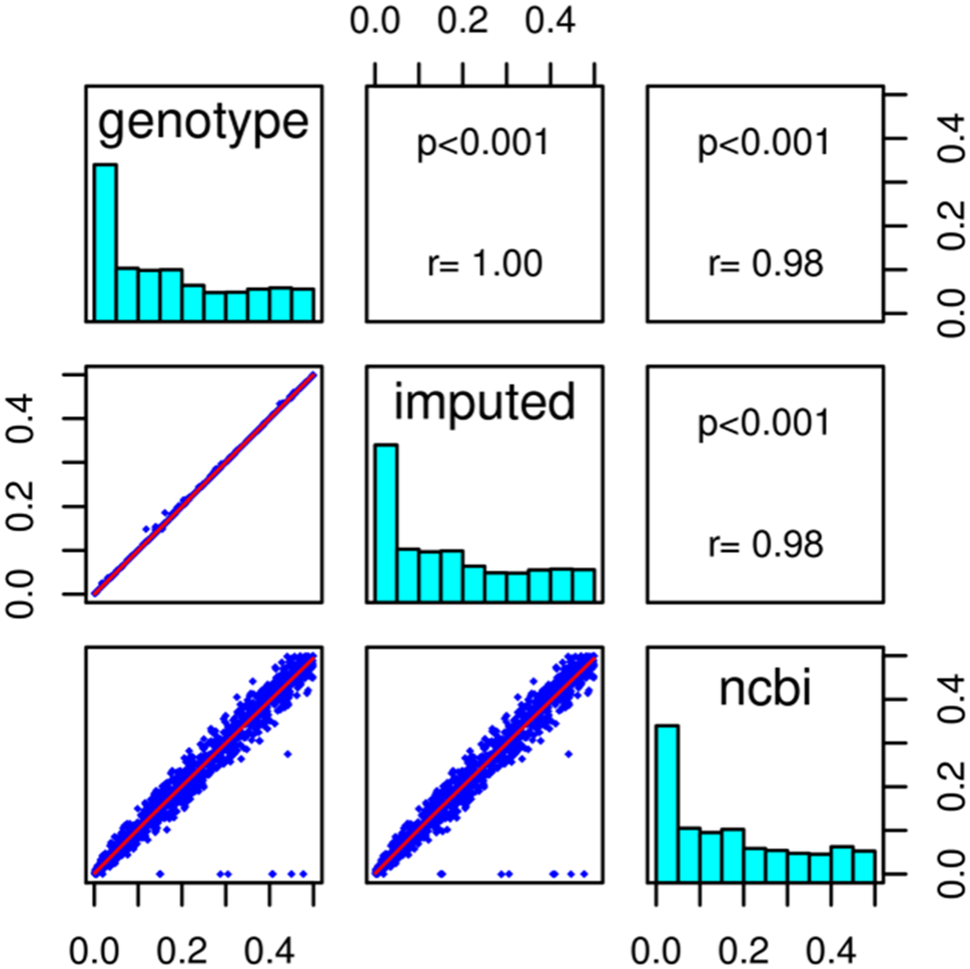
Correlations between the MAFs of genotyped, imputed variants and those of the NCBI dbSNP B137. In the absence of pre-filtration only 13 SNPs were not correlated with NCBI dbSNP B137.

Further investigation of the pair-wise correlation coefficient between MAFs of the genotypes and imputed variants revealed that the Spearman correlation was at its maximum value of 1 (Fig. 1, noQC), suggesting that the imputation reflected the original genotypes. Southam et al.^42^ previously reported a strong positive correlation between genotyping and imputation which they defined as reflecting a high degree of imputation accuracy. The genotyped SNPs that failed QC were also strongly correlated (r2 = 1) with the MAF of the imputed variants with or without pre-QC filtration, and also with their NCBI records. This suggests that in this case their exclusion was not essential. The SNP alleles and allele counts after imputation, with or without QC, were the same for heterozygotes and homozygotes (Supplementary Fig. S3).

We found no significant differences in the number of variants before and after QC (p-value = 0.945) nor in the overall frequencies (p-values = 0.512) using the Wilcoxon paired test, and the overall MAFs were identical (mean = 0.05, sd = 0.110). We divided the MAFs and NCBI registered variants (0–1E−04] – (1E0−4–1E−03] – (1E−03–1E−02] – (1E−02–5E−02] – (5E−02–1E−01] – (1E−01–5E−01] into six distinct classes for each imputed SNP genotype and found no outliers outside the lower and upper quantiles. Thus the classification for categorising the different MAFs was considered appropriate.

The differences obtained from the paired test for each category of allele frequencies, imputed with or without SNP QC, were not significant except for the null alleles, the second group (5E−04–1E−03] of very rare variants and the first group of rare variants (1E−03–5E−03] (Table 1).

QC had no effect on the number of monoallelic variants (3299 with QC and 3292 without QC), 2717 variants (82.5%) were common to each imputation round. In the absence of QC, the remaining 16% (526) were imputed after QC as very rare subclass I (1E−04–5E−04), 1.4% (46) as very rare subclass II (5E−04–1E−03) and 0.1% (3) as rare variants (1E−03–5E−03). The same percentages were observed when comparing the variants obtained by applying QC to those obtained without QC. The monomorphic variants in presence of QC that were imputed in absence of QC as very rare (subclasses I, II) and rare were also of 16%, 1.4% and 0.1%, respectively (Supplementary Fig. S4). Most changes in classes occurred between the MAFs of the monoallelic and very rare variants, due to the number of heterozygous counts below 1, as derived from probabilities generated by IMPUTE2. This suggests that some variants switched between these classes.

**Figure 2:**
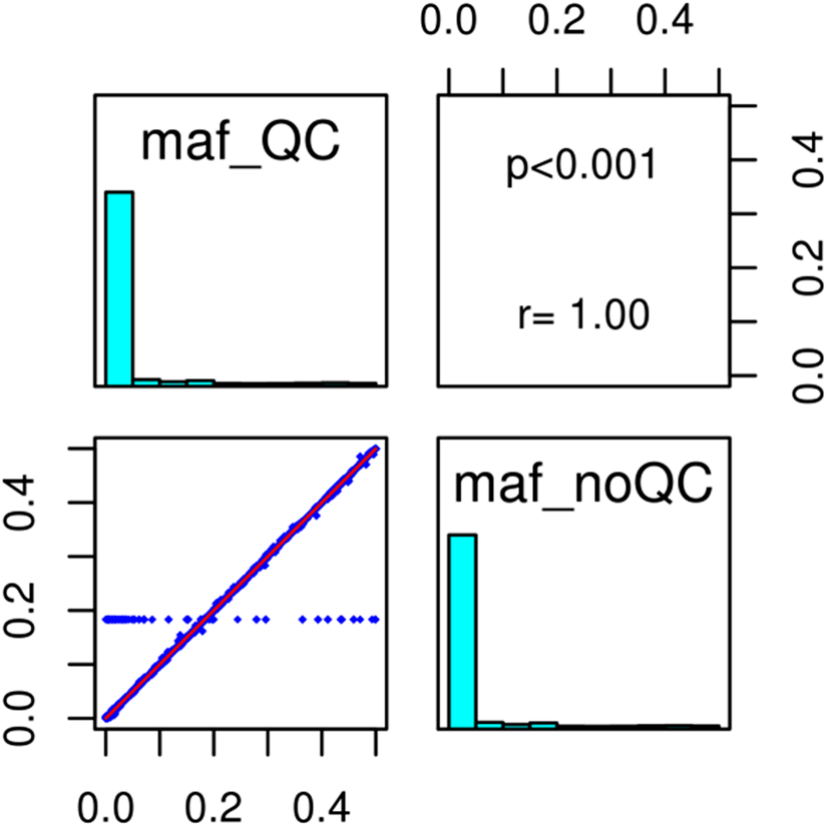
Correlation between the MAF imputed-only markers (non genotyped) with and without QC pre-filtration (44 unreliable structural variants included 12 monomorphic variants reported in dbSNP B137; 29 absent from dbSNP B137 and 3 with discordant alleles compared to NCBI).

We observed a high degree of correlation between the frequency of minor alleles obtained after imputation with or without pre-filtration. Only 44 structural variants (0.16%) showed discordance in their MAF. Without QC, their MAFs were identical (0.18223) but with QC, they varied widely (Fig. 2). Furthermore, they had incomplete alleles and name records in NCBI, or were described as monomorphic and 40 variants imputed different alleles before and after QC. We thus considered them as unreliable despite their information score of > 0.8. The public database served here has a further control.

### Comparing databases

Genotyped variants imputed with or without pre-filtration had a strong MAF correlation (r2 = 0.99) with the dbSNPB137.

**Figure 3:**
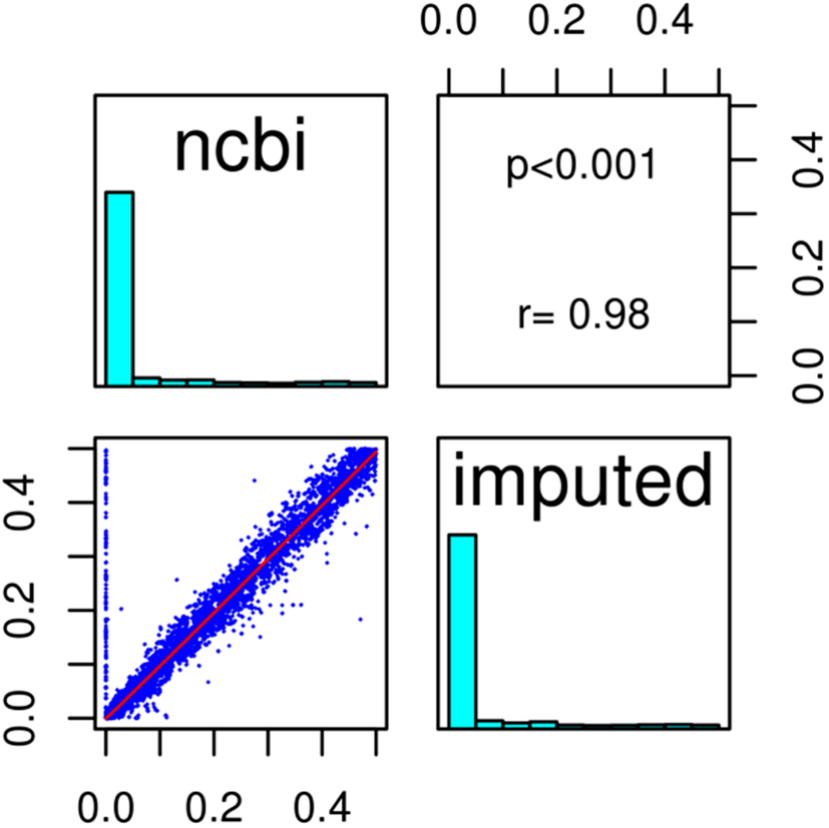
Correlations between the variant MAF in NCBI dbSNP137 and the MAF after imputation without pre-filtration: 163 variants (imputed-only) were recorded in NCBI with null allele frequencies (18 imputed with MAF = 0 and information score < 0.3; 145 mostly structural variants uncorrelated with their imputed MAF).

For the imputed-only variants, with or without QC, only 163 were registered with null minor allele frequencies in dbSNP B137, including 18 variants detrimentally imputed with null MAFs (Fig. 3). The MAFs of the remaining 145 not fully documented in dbSNP B137 (personal communication) were thus uncorrelated with the NCBI MAFs (Supplementary Fig. S5) and 80% (0.44% in total) were imputed as structural. The majority had an information score above 0.8. Similar values were obtained when QC was applied. Additionally, more than 50% of the 145 variants were recently found in *Ensembl* GrCh38.p10 with frequencies between 2E−04 and 0.45^51,52^.

We found that 13 genotyped rare to frequent variants absent from the reference haplotype (Fig. 1) were efficiently imputed (information-impute2 score = 1) without pre-filtration and could thus be kept for downstream analysis. Independently, we also identified 18 dual imputations (i.e. 36) when a structural variant and SNP were present at the same locus.

### Imputation quality

**Figure 4:**
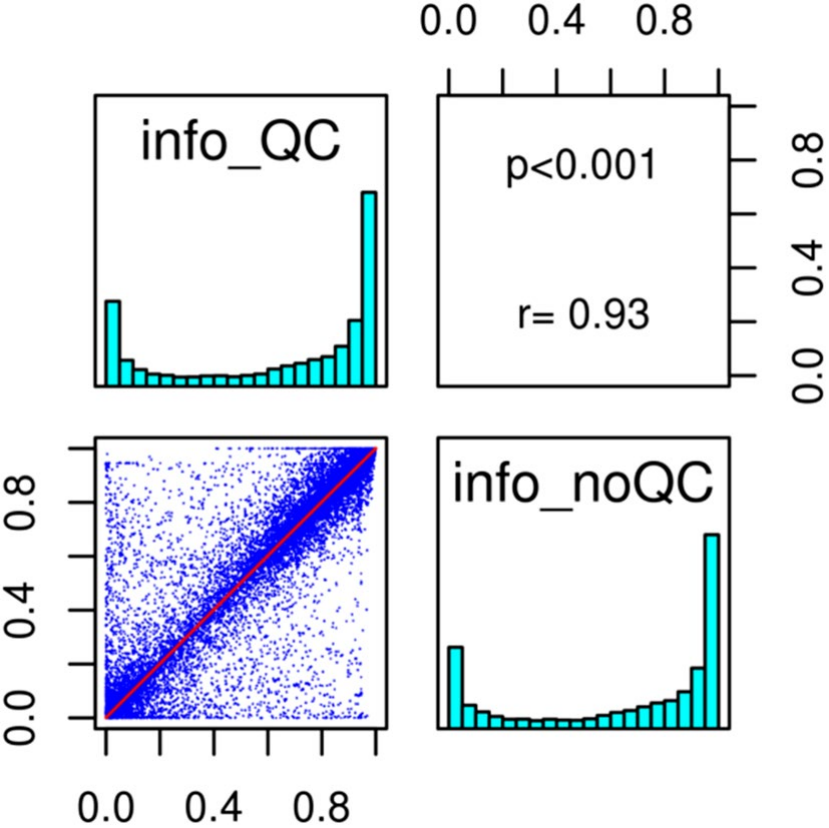
Correlation between information-impute2 scores obtained after QC pre-filtration (info_QC) and no QC variant filtration (info_noQC).

We applied the paired test to each subclass of all imputed variants and found a significant group effect between the information imputation scores observed before and after quality control (Table 2). Thus the same variant did not necessarily produce the same information score with and without QC, although the overall mean imputation quality scores were similar before and after SNP filtering (Table 2). This difference can also be seen in Fig. 4 where the information scores are spread across each part of the regression line with a Spearman coefficient of only 0.93.

**Table 2:** One tail Wilcoxon-paired signed rank test comparing information before (no QC) and after QC (QC).

maf categories	Info no QC		Info QC		Wilcoxon paired test	Info no QC - info QC
	Mean	sd	Mean	sd	Info right tail p-value	Pseudomedians
0–0	1.70 E−02	2.60 E−02	1.60 E−02	2.60 E−02	1.00	− 0.00995
1E−04-5E−04	3.10 E−01	3.10 E−01	3.00 E−01	3.07 E−01	3.30 E−04 ***	0.00360
5E−04-1E−03	6.10 E−01	2.60 E−01	6.20 E−01	2.60 E−01	3.48 E-16 ****	0.01350
1E−03-5E−03	7.70 E−01	1.97 E−01	7.60 E−01	1.98 E−01	< 2.20 E-16 ****	0.00650
5E−03-1E−02	8.80 E−01	1.30 E−01	8.70 E−01	1.30 E−01	< 2.20 E-16 ****	0.00740
1E−02-5E−02	9.40 E−01	9.50 E−02	9.40 E−01	9.80 E−02	< 2.20 E-16 ****	0.00198
5E−02-1E−01	9.70 E−01	5.20 E−02	9.70 E−01	5.60 E−02	9.50 E−03 *	0.00580
1E−01-5E−01	9.90 E−01	3.90 E−02	9.90 E−01	3.40 E−02	< 2.20E-16 ****	0.00969
0-5E−01	6.72 E−01	3.68 E−01	6.60 E−01	3.67 E−01	< 2.20E-16 ****	0.00206

Significant differences in information scores were found in each class except the first category as null alleles hold no information.

Right tail levels of significance **** < 0.00005, *** < 0.0005, ** < 0.005, * < 0.025.

The differences between the two distributions were at least equal to their pseudomedians (pseudomedians were obtained from the differences between the distributions before and after QC).

*Info* information imputation score, *maf* minor allele frequency, *sd* standard deviation, *QC* quality control.

Pre-filtration improved the quality of the genotyped SNPs (Supplementary materials) but decreased by 17.5% the number of variants available for imputation. However, as stated above, the genotyped SNPs that failed initial QC were better imputed (information-impute2 score = 1) than those excluded prior to imputation (information-impute2 score = 0.4–1), and the information score above 0.3 was maintained in each case. Further, in conditions without pre-filtration compared with pre-filtration, the SNP added confidence to the imputation. The one-sided Wilcoxon paired test showed significantly greater information without SNP pre-filtration than with pre-filtration, these differences were observed at the alpha-level < 5E−04 in all classes except for the common variants (Table 2).

### Post-filtration

**Figure 5:**
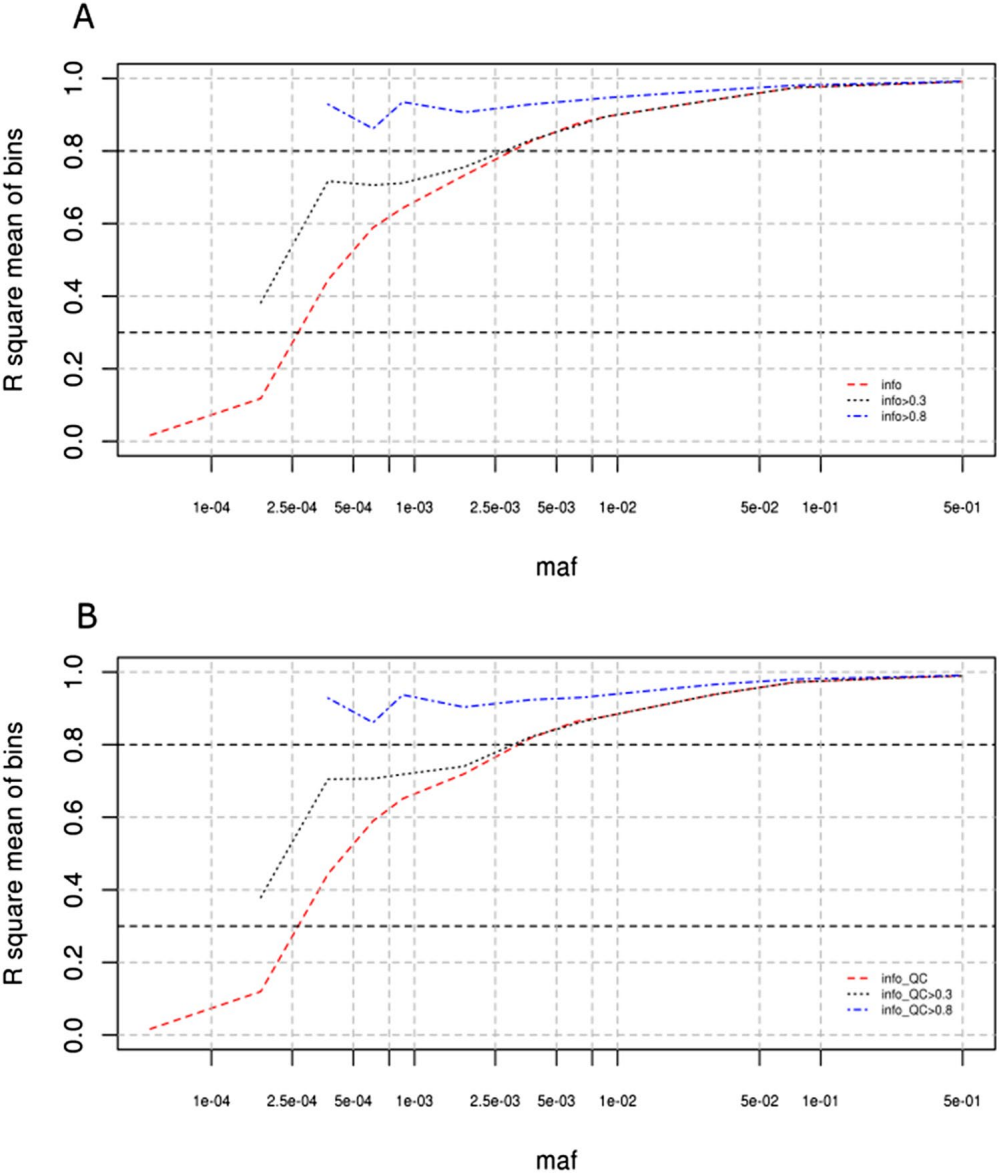
**(a)** Absence of QC: r-square (info-impute2 metric) mean of bins for variants according to the log(maf). Under 3 different post-filtration conditions (absence of post-filtration; post-filtration at an information score > 0.3; post-filtration at an information score > 0.8). Each middle bin is joined by a coloured dotted line. From 0.01 MAF, the information score tends towards the maximum information even in the absence of pre-filtration. **(b)** After QC pre-filtration: r-square (info metric) mean of bins for variants vs log(maf). Each middle bin is represented by a coloured dotted line (absence of post-filtration; post-filtration at an information score > 0.3; post-filtration at an information score > 0.8).

Regardless of whether or not QC was applied prior to imputation, the total number of variants was reduced by almost a quarter, and by half for post-filtration, based on imputation scores of 0.3 and 0.8 respectively. This increase from 0.3 to 0.8 caused a 1.8 fold decrease in the number of variants for MAF < 0.01. Filtering using the information scores of 0.3 or 0.8 following SNP quality control mirrored the filtration without QC (Supplementary Fig.S6), i.e. both curves overlapped (Fig. 5).

The presence or absence of pre-filtration had little impact on post-filtration of imputed variants at MAF > 0.01 (Fig. 5, Supplementary Fig. S6) as their mean quality score was already over 0.8**.** The mean of bins for information score within the frequency range 5E−04–5E−03 was further improved when filtering was above 0.8 (Fig. 5). Without post-filtration, we observed for both runs an average score higher than 0.7 in the rare variant subclass (1E−03–5E−03) and just over 0.6 in the very rare variant subclass (5E−04–1E−03), showing that the information decreased with the MAF (Table 2).

Post-filtration of the data removed additional variants in the very rare classes. Downstream filtration at 0.3 showed that at a MAF of 5E−04, the average information score obtained after applying this threshold was closer to 0.7 (Fig. 5). When the threshold was raised to 0.8, the mean information score at a MAF of 5E−04 increased to above 0.8 (Fig. 5), demonstrating that certain variants at 5E−04 had been eliminated.

The number of very rare variants in the MAF subclasses between 2.5E−04 and 5E−04 decreased by almost half when the quality score was incremented from 0.3 to 0.8, in the presence or absence of pre-QC filtration. Below a mean MAF of 0.001, the ratio for the number of SNVs decreased 2.5 fold when post-filtration was incremented from a less conservative (0.3) to a more conservative (0.8) quality score. Thus, applying a lower information threshold allowed more very rare variants to be kept but the variants were of lower quality*.* Therefore, it may be of interest to use post-filtration at 0.3 in order to keep MAF variants < 0.001 (e.g. 5E−04) and to use a more stringent cut-off to maintain SNVs above 0.001. The sd of the quality score also decreased as the MAF increased. The sd was less densely spread around the mean when filtering with a threshold of 0.8 rather than 0.3. The quality of the data improved when the filtration threshold stringency was raised, but this also lowered the number of variants (Supplementary Fig. S7).

In the absence of post-filtration, the mean quality score for a MAF of 7.5E−04 was 0.7 in the range of 0.32–0.85, representing between one and two heterozygotes. Therefore, filtering MAFs between 5E−04 and 1E−03 (7.5E−04) should produce an imputation score above 0.3 (Supplementary Fig. S7). A frequency of 7.5E−04 includes MAFs that are just above the frequency of the sample set representing just over one imputed heterozygous individual (i.e. 1.5) and ensuring better confidence in the probabilities of the number of estimated alleles. The MAF for a sample set is seldom achieved during genotyping for GWAS and fine-scale mapping studies, especially for homozygous rare variants. Genotyping studies of 1000 individuals would not have captured either homozygous or heterozygous rare variants with pre-filtration at a MAF < 0.01.

**Figure 6:**
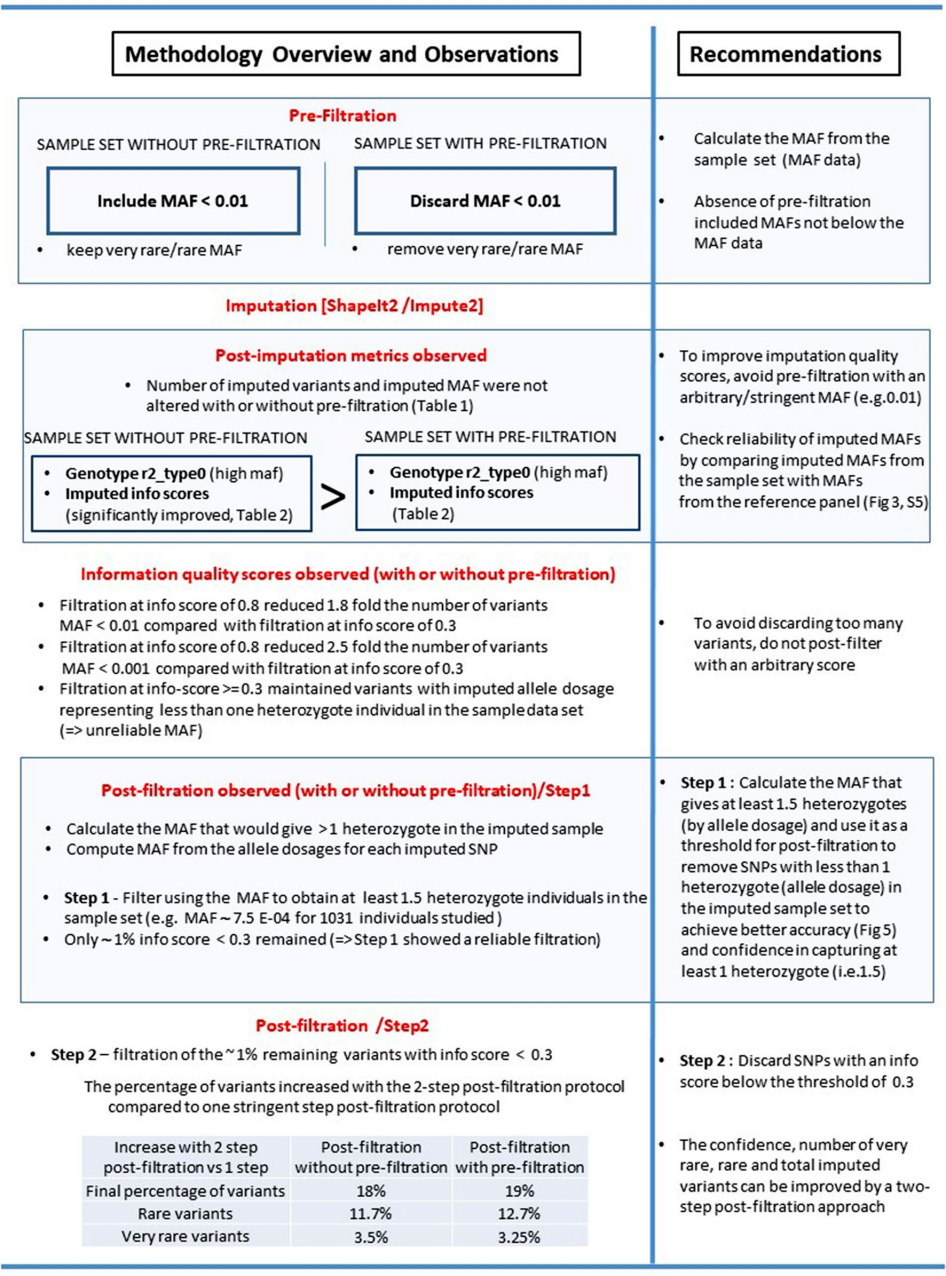
Methodology overview. After imputation a significant amount of variants are discarded by current practices. Some of these variants may provide important insight for determining disease risk. Here we investigate the effect of filtration strategies on the profiles of imputed variants and provide recommendation to improve the imputation quality and to reduce the amount of variants discarded.

When we performed pre-filtration followed by an initial post-filtration at a MAF of 7.5E−04, only 1.24% of variants remained with a low imputation quality score (< 0.3), similar to the 1.33% obtained without pre-filtration. As expected, the mean for the class (7.5E−04 and 1E−03) reached an information score close to 0.7, i.e. 0.64 (sd = 0.26). The second step required only filtering out ~ 1% remaining variants with a score below 0.3 to enhance confidence (Fig. 6). When this 2-step protocol was compared with a single-step protocol using post-filtration at a stringent accuracy of 0.8, i.e. 18% (no-QC) and 19% (with QC) respectively, less variants were eliminated: i.e. for very rare variants, 3.5% (no-QC) and 3.25% (with QC); for rare variants, 11.7% (no QC) and 12.7% (with QC). Hence this is a useful gain.

## Discussion

Imputation can create dense maps that can feasibly be used to refine loci linked to disease genes. In this study we ran SHAPEIT combined with IMPUTE, programs that have been commonly used for imputation with the 1000GP in fine-mapping of small regions previously missed in GWAS^53,54^.

Imputation from the reference panels allowed searches for variants forming haplotypes of identical sequences to those of genotyped individuals in order to impute their missing variants^14^. Thus, for comparison of variants and MAF frequencies, individuals to be imputed were of similar number and ethnic background to the reference genome. Due to inherent sample effects, rare variants may be absent from the reference panel^55,56^. This effect can be compensated for by the presence of populations from different demographic regions where these rarer variants are more common^14,55,56^. Using the same number of subjects as the 1000GP, we compared imputed variants with and without pre-filtration of low quality genotyped SNPs. The size of the region, similar to the size of a fine mapping region, allowed us to manually curate the SNPs and to use the same seed for reproducibility of the imputation runs. The 2 Mb region in terms of counts, density and MAFs of SNPs was representative of the 2 Mb bins of Chr20 and the whole genome (Supplementary Fig.S1, S2, Table S1).

IMPUTE2, being map-dependent, uses the reference dataset to enable the software to fill in gaps. After pre-filtration, the 17.5% of SNPs previously eliminated were fully recovered. Unlike previous studies, we did not observe a detrimental effect on imputation with the 1000GP when pre-filtration was not applied, probably due to improvements in the HapMap 3 reference set^3^. Thus it is possible to decrease the MAF in the pre-filtration criteria and still achieve full imputation. It has been reported that the absence of variant pre-filtration with the GATK variant quality score recalibration (VQSR) does not reduce the numbers or performance of imputation on sequencing data^57^.

SHAPEIT is designed to perform well when missingness is low^43^, which suggests that the absence of filtration may improve imputation performance. This seems to be consistent with findings observed in our experiments performed without pre-filtration, and in this study the SNPs that failed QC showed a reliable imputation. Their allele calls were also identical to those obtained after pre-QC filtration with a similar number of allele counts (Supplementary Fig. S3). Further, in the absence of pre-filtration, the imputation was improved; the r2_type0 of the genotypes SNPs was significantly increased for the most frequent MAFs 0.01–0.05 (p = 1E−06, one-tailed) and the information scores were significantly higher in all classes of MAFs (Table 2).

We observed a good correlation of MAF after imputation with or without QC (Fig. 2) and only very low subclasses of MAF were found to be significantly different due to the closeness of the low allele counts (Table 1). QC-filtered SNPs that were absent from the reference genome were not imputed. Imputation quality could also be observed by comparing the MAFs of imputed variants with those of public databases such as NCBI, used as a control. We also showed that a high information score did not guarantee a fully documented variant in dbSNP. The correlated MAFs also revealed variants mislabelled with null alleles in dbSNP B137 (Supplementary Fig.S5). After imputation, monomorphic variants have been reported^58^ to be present in reference panels, including GoNL^59^. They may also be present in study data or when the samples do not share enough segments of common ancestry with the reference panel.

Sampson et al. demonstrated that increasing the sample size using the 1000GP panel improved imputation performance^60^; in our case the size of the cohort was above 1000. Compared to imputations using panels with less individuals, a gain in very rare and rare imputed variants was found with the 1000GP^3,42^.

Difficulties were encountered with previous panels when attempting to impute rare variants (MAF < 0.5%) as their performance remained lower compared to that of the more common variants^61^. With IMPUTE2, Deelen et al. found that variants with a MAF of 0.05–0.5% would remain with a mean imputation accuracy below 0.8 with samples of the same ethnicity as the GoNL Panel^62^*.* Other authors have performed an initial QC variant filtration and reported that below a MAF of 0.05 the information score decreased to under 0.8, while it increased to above 0.8 in higher MAF categories^63^. Pistis et al.^12^ tested IMPUTE and finally chose a score > 0.7 rather than 0.3–0.4 to ensure that only well-imputed variants were kept. We found that a high accuracy score of 0.8 removed too many variants and, as a result, we had to apply a lower filtration accuracy score to keep very rare frequency variants < 0.001. A mean information score > 0.8 was reached when the mean MAF was > 2.5E−03 (i.e. 5E−03), in the presence or absence of post-filtration, which excluded mainly the very rare and some rare variants (Supplementary Fig. S7).

Increasing the accuracy threshold to above 0.8 has been shown to empirically reduce statistical association with the Armitage trend test^64^ and decrease the number of variants. It has been suggested that excluding too many variants decreases the power of association tests whilst in meta-analyses, it can lead to a loss of information^40,41^. However better imputation accuracy could also mean improved statistical power in association analyses^65^. Currently there is no consensus on a post-filtration imputation threshold to ensure reliable downstream analysis^12^.

In a two-step imputation approach, Kreiner-Moller et al. used successively an in-house reference set and the 1000GP panel. The second imputation step improved accuracy in the absence of QC or when only filtering markers with a quality threshold of 0.3 compared to markers with a threshold of 0.8^11^. These authors suggested that eliminating too many markers in the first step leads to a reduction in quality during the second imputation step. Other authors also advocate against excluding too many SNPs as they could potentially provide supplementary information to impute variants even with low correlation^39^. Moreover, if too many SNPs are missing, the haplotypes may be incomplete^38,39,66^. Improvements in accuracy have also been reported when genotype information is available for markers tightly linked to those being imputed^3^.

In association studies, pre-QC based imputation with extensive post-filtration using the 1000GP panel may be being performed at the expense of removing causal variants. We would suggest instead that single-point association studies that previously removed SNPs by applying an imputation information score > 0.8, should be reanalysed using a score of 0.3–0.8 with an imputed MAF threshold representing at least one heterozygous individual from the dataset. As each marker at this MAF represents at least one individual, this approach will improve the confidence of post-filtration (Supplementary Fig. S7). This method could also improve association analyses as it increases the number of very rare and rare variants.

For future GWAS and fine-scale mapping, we therefore recommend calculating the MAF of the sample set studied rather than filtering the genotypes prior to imputation with an arbitrary MAF of 0.01 or 0.005. We advise primarily not to pre-filter below the MAF of the sample set. We then suggest applying the following two-step post-filtration method: (1) Filter slightly above the MAF of the samples to achieve better accuracy; (2) Remove any remaining variants with an imputation score below 0.3 so that only reliable variants with an information score between 0.3 and 0.8 are retained and can be flagged prior to analysis (Fig. 6). Previous reports^67,68^ have mentioned the possible role of MAFs on GWAS and imputation, which supports our findings. In our experience, this approach mainly improves imputation confidence above the score of 0.3.

In addition, genotype imputation approaches which use arrays or low coverage genotyping-by-sequencing (GBS) remain cost-effective compared to whole genome sequencing ^55,69–71^. Also, other reference panels can be used with the methodology since the MAF for filtration can be calculated from the studied sample data. Panels such as HRC^20^, UK10K^21^ or TOPMed^22^ provide more imputation accuracy and would therefore enhance the methodology that we present here.

## Conclusion

SNP pre-filtration above the minor allele frequencies of heterozygotes in the sample set should be avoided to reduce imputation information loss. The confidence and number of very rare and rare imputed variants can be improved by using the two-step post-filtration method presented above.

## Supplementary Information

Supplementary Information.

## Data Availability

The datasets used in the present study are available from https://mathgen.stats.ox.ac.uk/genetics_software/shapeit/shapeit.html, ftp://ftp.1000genomes.ebi.ac.uk/vol1/ftp/technical/working/20130606_sample_info/20130606_sample_info.xlsx, ftp://ftp.1000genomes.ebi.ac.uk/vol1/ftp/phase1/analysis_results/shapeit2_phased_haplotypes/, https://mathgen.stats.ox.ac.uk/impute/data_download_1000G_phase1_integrated_SHAPEIT2_16-06-14.html. The data generated and analysed in this study are included in this article and in the Supplementary Information files. The individual files are also available on request from the corresponding author.
