# Publisher Correction: Impact of pre- and post-variant filtration strategies on imputation

**DOI:** 10.1038/s41598-021-99038-w

**Published:** 2021-09-22

**Authors:** Céline Charon, Rodrigue Allodji, Vincent Meyer, Jean‑François Deleuze

**Affiliations:** 1 CEA Paris-SaclayInstitut François Jacob, Centre National de Recherche en Génomique Humaine, 2 rue Gaston Crémieux, Evry, 91057 France; 2 Radiation Epidemiology Group CESP, Inserm Unit 1018, Gustave Roussy Université Paris Saclay, 114 rue Edouard Vaillant, Villejuif, 94805 France

Correction to: *Scientific Reports* https://doi.org/10.1038/s41598-021-85333-z, published online 18 March 2021

The Supplementary Information file published with this Article contained an error, where the author Rodrigue Allodji was incorrectly affiliated with ‘Institut Gustave Roussy, Dept. Biostatistics & Epidemiology, Villejuif, France’. The correct affiliation is listed below:

Radiation Epidemiology Group CESP, Inserm Unit 1018, Gustave Roussy Université Paris Saclay, 114 rue Edouard Vaillant, Villejuif, 94805, France

This error has now been corrected in the Supplementary Information file that accompanies the original Article.

