# Supplementary material for: Impact of pre- and post-variant filtration strategies on imputation
Source: Sci Rep. 2021 Mar 18;11:6214. doi: 10.1038/s41598-021-85333-z (PMC7973508; doi:10.1038/s41598-021-85333-z)
Supplement: Supplementary file 1 — Supplementary Information. [file 41598_2021_85333_MOESM1_ESM.pdf]

Supplementary information files

Impact of pre and post variant filtration strategies on imputation

Céline Charon[1,*], Rodrigue Allodji[2], Vincent Meyer[1] and Jean-François Deleuze[1]

[1] CEA Paris-Saclay, Institut François Jacob, Centre National de Recherche en Génomique Humaine, Evry, France
[2] Radiation Epidemiology Group CESP, Inserm Unit 1018, Gustave Roussy Universite Paris-Saclay, 114 rue Edouard Vaillant, Villejuif, France

Materials and methods

Quality control of the genotypes was performed with PLINK (v 1.07)[72,73] prior to pre-phasing with SHAPEIT2 and imputation. The MAF was determined with SNPTEST v2.5.2[34]. A total of 0.11% of the 1,760 genotyped SNPs were stratified as very rare frequency variants (5E-04-1E-03), 11.70% as rare (1E-03-1E-02), 19.10% as low (1E-02-5E-02), 10.51% as common (5E-02-1E-01) and 58.58% as high (1E-01-5E-01) frequency variants. Autosomal SNPs with successful call rates < 99%, (Hardy Weinberg Equilibrium) HWE < 1E0-6 and MAF < 1% were excluded during QC. SNPs with a low HWE corresponded to common and high allelic frequencies, and call rates below 99% included only 5 SNPs with rare frequencies. In total, 308 SNPs from 1,760 variants did not pass the test criteria. After removal of these variants, QC was performed on individuals with a genotyping success rate of 97% for one individual and 99% for another. Before removal of the variants, the success rate was : 97% for 2 individuals, 98% for 3 individuals and 99% for 5 individuals. Finally, as the lowest success rate remained between 97%-99% for 4 individuals before and after elimination of the variants, we kept all individuals in order to maintain similar conditions using SHAPEIT2 and IMPUTE2. Thus we were able to investigate the effect of missing variants only.

We built an SQL database to upload the NCBI reference dbSNP of the human build 137 based on GCRCh37 assembly 37.4 (dbSNP B137). After imputation, certain imputed SNPs were curated manually, i.e. those that had changed names between the imputation and the NCBI repositories. Curation also allocated the correct rs numbers from the NCBI database from duplicates, unreferenced SNPs, i.e. missing rs number from dbSNP, merged SNPs or rs numbers that had changed records from polymorphism to deletion at the same position. After imputation, we categorised the variant frequencies as desccribed in materials and methods and compared their MAF before and after QC and queried the NCBI database for further control, since the samples (1,031) and dbSNPs (1,089) were of similar size and population origin.

Table S1. Mean of SNP counts and densities every 2 Mb per autosome and standard deviation (sd)

chromosomes	mean SNP counts/2Mb	sd	mean SNP densities/2Mb	sd
1	25188.27	4595.06	12.59	2.30
2	26144.87	5096.45	13.07	2.55
3	26907.28	4242.71	13.45	2.12
4	27724.94	3697.88	13.86	1.85
5	26798.82	4626.74	13.40	2.31
6	27099.38	4592.27	13.55	2.30
7	26651.72	5780.50	13.33	2.89
8	28921.27	7873.46	14.46	3.94
9	25307.11	9850.80	12.65	4.93
10	27074.66	4679.54	13.54	2.34
11	27269.69	2955.49	13.63	1.48
12	26261.54	4023.74	13.13	2.01
13	26663.35	2750.64	13.33	1.38
14	26909.40	4166.96	13.45	2.08
15	25925.31	6619.92	12.96	3.31
16	28595.73	11268.50	14.30	5.63
17	24559.15	5636.77	12.28	2.82
18	26870.18	5548.14	13.44	2.77
19	26057.30	6386.25	13.03	3.19
20	25777.06	6876.64	12.89	3.44
21	24889.90	10664.19	12.44	5.33
22	26404.06	6047.25	13.20	3.02

The number (27,090) and density of SNPs (13.545/kb) on the 1000GP1 for the 2 Mb region studied are within the sd of the average counts and the density of SNPs every 2 Mb of the 1000GP1 in the genome.

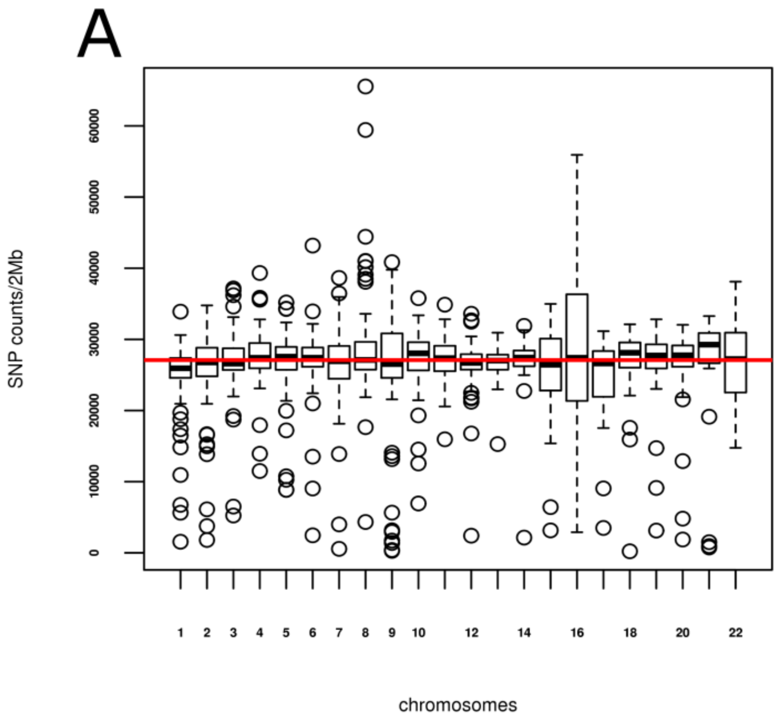

Fig.S1 (A) SNP counts every 2 Mb per chromosome. Each chromosome from the 1000GP1 was sequentially split every 2 Mb, resulting in 1,396 bins that covered the genome. The number of SNPs within the 2 Mb region studied, on the 1000GP1 (red line : 27,090), lies at the median of the counts from each autosome and Chr 20 every 2 Mb or well within their interquartile ranges (IQRs). 2% of the genome was not covered by any SNPs, 58Mb i.e. 29 bins from 1396 bins of 2Mb : Chr 1 : 122-142 Mb, Chr 4 : 50-52 Mb, Chr 8 : 44-46 Mb, Chr 9 : 48-64 Mb, Chr 10 : 40-42 Mb, Chr 11 : 52-54 Mb, Chr 16 : 36-46 Mb, Chr18 : 16-18 Mb, Chr 21:120-122Mb

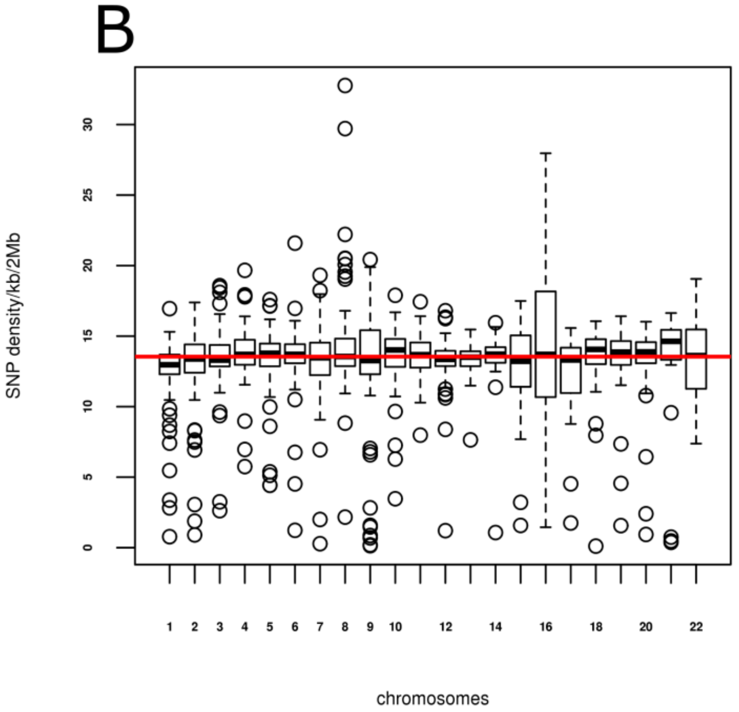

Fig.S1 (B) SNP densities every 2 Mb per chromosome. Each chromosome from the 1000GP1 was sequentially split every 2 Mb, resulting in 1396 bins that covered the genome. The density of the 2 Mb region studied, on the 1000GP1 (red line : 13.545 SNP/kb) is close to the median and within the IQRs of the SNP densities every 2Mb of the genome per chromosome including Chr 20. 2% of the genome was not covered by any SNPs, 58Mb i.e. 29 bins from 1396 bins of 2Mb : Chr 1 : 122-142 Mb, Chr 4 : 50-52 Mb, Chr 8 : 44-46 Mb, Chr 9 : 48-64 Mb, Chr 10 : 40-42 Mb, Chr 11 : 52-54 Mb, Chr 16 : 36-46 Mb, Chr18 : 16-18 Mb, Chr 21 : 120-122 Mb.

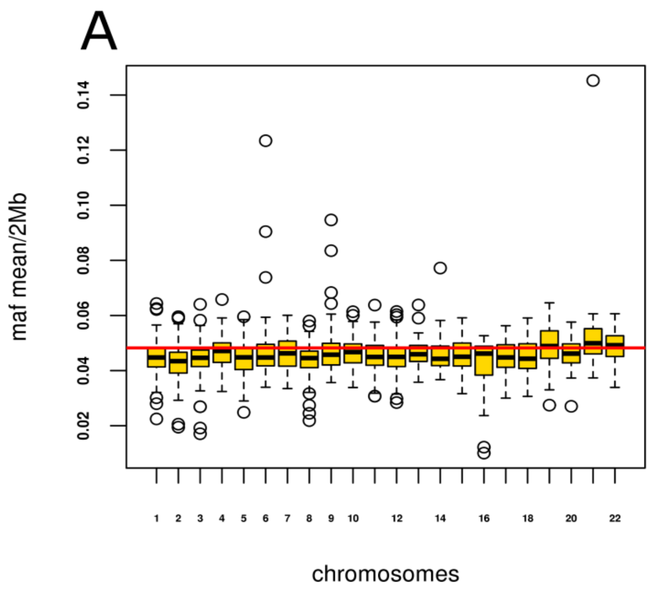

Fig.S2 (A) Mean scores of the MAF every 2 Mb bin per autosome on the 1000GP1 are shown in the boxplots: Red line : the average MAF on the 1000GP1 of the 2 Mb region studied (0.0482) is within the midspread (IQR) of the mean of the MAFs every 2Mb per autosome, and close to the IQR of chromosome 2 (0.0400-0.0465) and chromosome 8 (0.041-0.0471). MAFs undefined by NCBI were not considered.

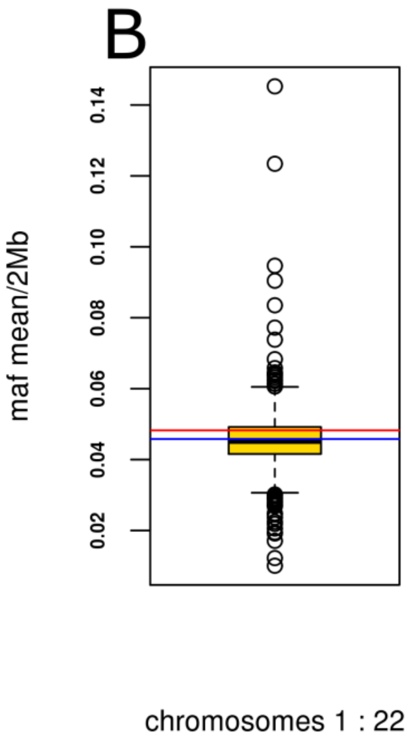

Fig.S2 (B) The 2Mb bins from figure S2A of the 1000GP1 autosomes are grouped together in the boxplot. The average MAF for the 1000GP1 of the region studied (red line : 0.0482) is within the midspread (IQR) of the 1367 mean scores of 2 Mb bins in the whole 1000GP1 genome (IQR : Q1 = 0.0415, Q3 = 0.0492). The pooled average of all MAF/2Mb of chr 20 (blue line : 0.0457) is also close to the median from the group of means every 2 Mb of the whole 1000GP1 genome (black line in boxplot : 0.0452)

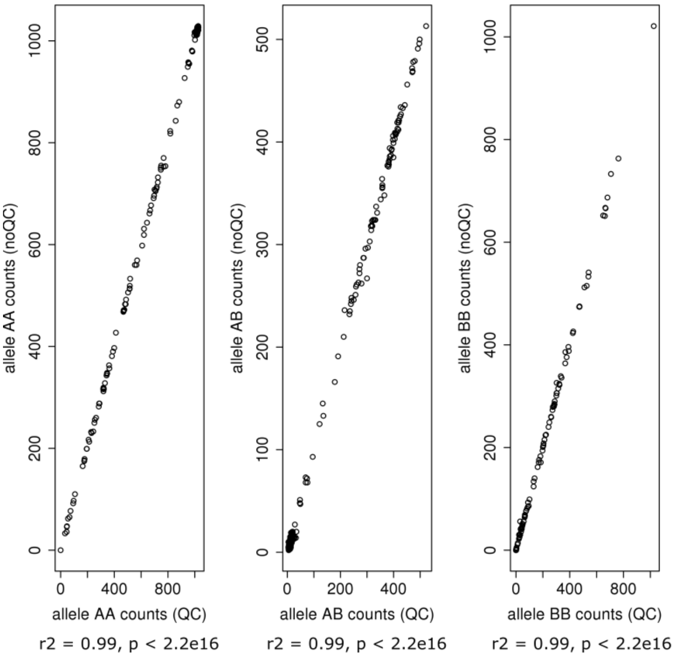

Fig.S3. SNPs that did not pass QC criteria: Allele counts of SNPs obtained after imputation in the absence of QC vs allele counts obtained with QC. The counts for the homozygous alleles were the same after imputation with pre-filtration of variants (QC) and without pre-filtration (no_QC), as shown by the r^2 close to 1. The same was observed for the heterozygous alleles. Their information score was > 0.3 (0.4-1) (results not shown).

Alleles AA: homozygous AA, allele AB: heterozygous AB, allele BB: homozygous BB.

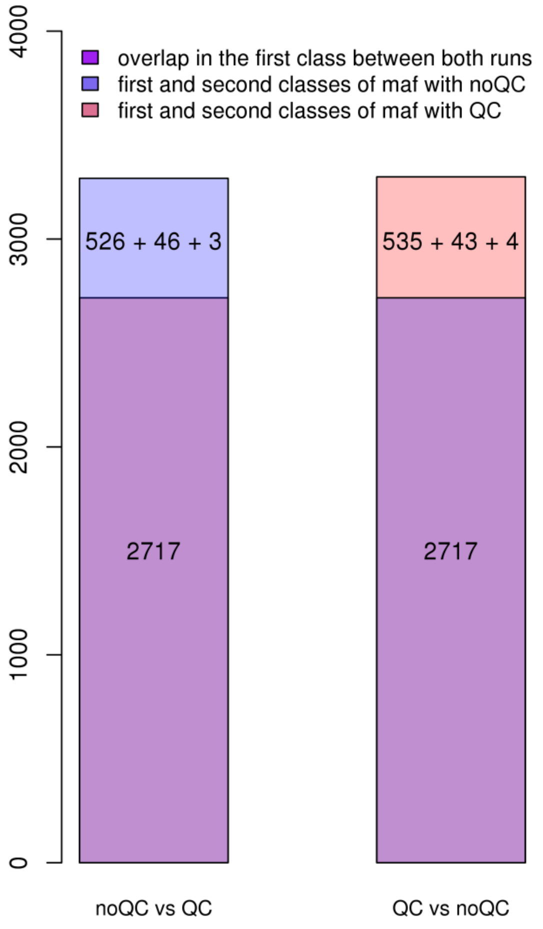

Fig.S4. Comparison of imputed variants: The number of monomorphic variants was similar with QC (3299) and without QC (3292), with 2717 of the same variants overlapping in both runs. 526 monoalleles imputed without QC were re-imputed withQC as very rare (0.0001-0.0005), while 535 monoalleles imputed with QC also reached a rare MAF (0.0001-0.0005) without QC. 46 and 3 monomorphic variants respectively were imputed as very rare (0.0005-0.001) and rare variants (0.001-0.005) without QC. After QC, these same markers also showed as very rare (0.0005-0.001) variants and rare variants (0.001-0.005) in the absence of QC.

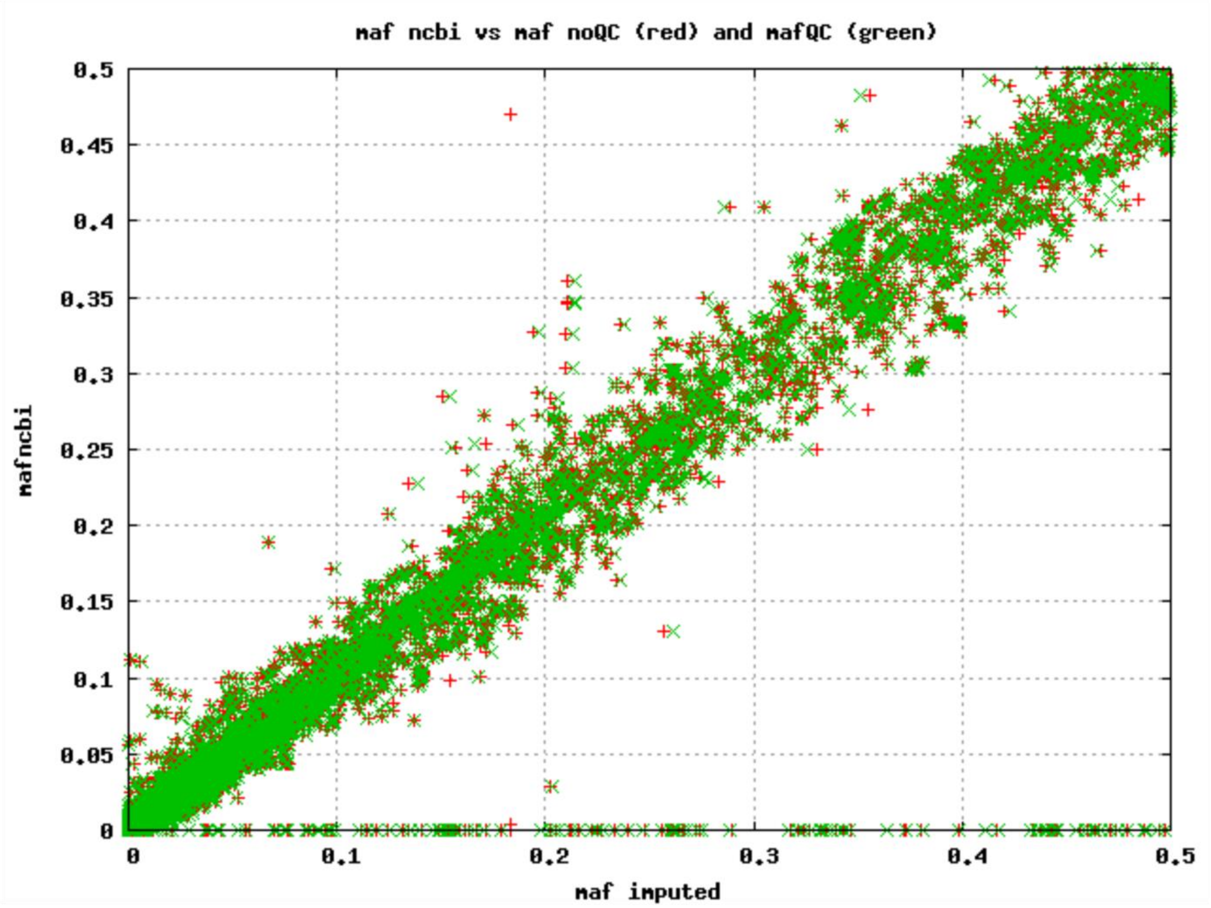

Fig.S5. Imputed variants either filtered (with QC) or unfiltered (without QC) vs NCBI show similar profiles. Positive linear correlations are shown between the NCBI MAF and the MAF imputed with QC pre-filtration ($r^2 = 0.98$, $p < 2.2e-16$) (green) and without QC pre-filtration ($r^2 = 0.98$, $p < 2.2e-16$) (red). Only 145 variants (0.52%) were recorded in NCBI dbSNP B137 with null MAF, and the majority were also reliably imputed with and without QC. To date, more than 50% of these 145 variants have been recovered as non-monomorphic in *Ensembl* GrCh38.p10, which is now based on 2,500 individuals.
“no QC” = imputed variants in the absence of QC pre-filtration, “QC” = imputed variants after QC pre-filtration

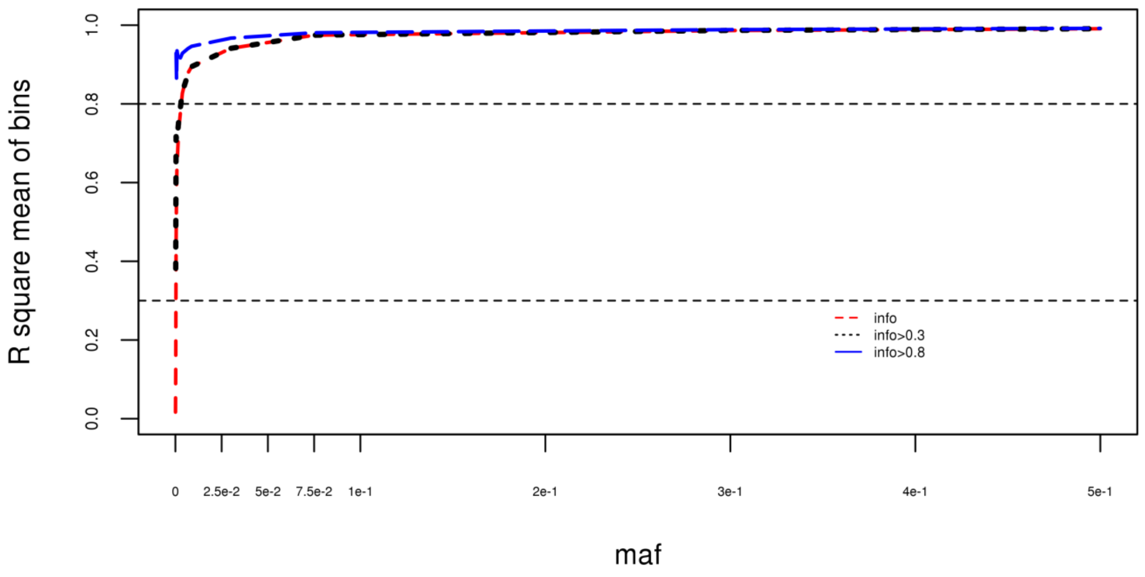

Fig.S6. Absence of pre-QC filtration: r-square (info-impute2 metric) mean of bins for variants MAF [0,0.0001] (null) (0.0001,0.00025] (0.00025,0.0005] (0.0005,0.00075] (0.00075,0.001] (very rare), (0.001,0.0025] (0.0025,0.005] (0.005,0.0075] (0.0075,0.01] (rare), (0.01,0.05] (low), (0.05,0.1] (common) and (0.1,0.5] (high). Each middle bin is represented by a coloured dotted line: absence of post-filtration (red dotted line); post-filtration at info 0.3 (black dotted line); post-filtration with info > 0.8 (blue dotted line).

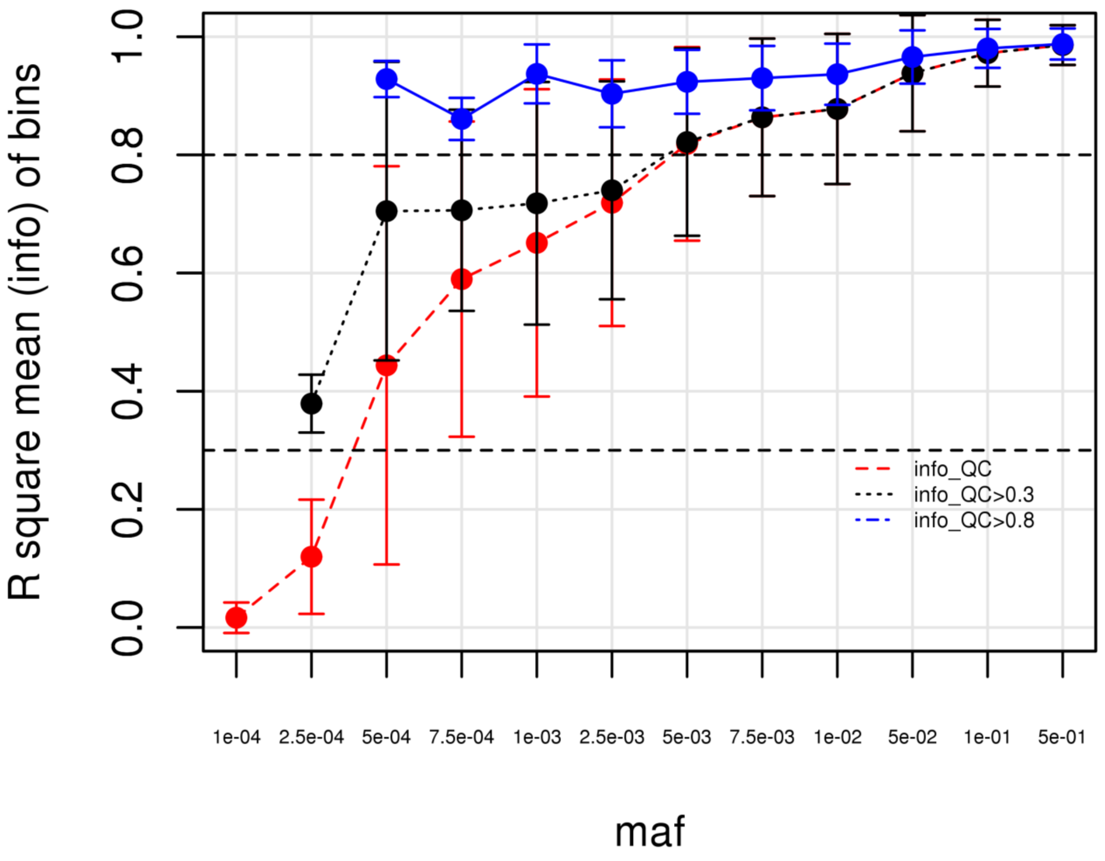

Fig.S7. Information scores are given for each middle MAF bin, with the standard deviation (sd) of the mean (vertical bars) in 3 different conditions after QC: (1) SNP QC without post-filtration (red); (2) SNP QC with post-filtration on information at 0.3 (black); (3) SNP post-filtration on information at 0.8 (blue). SNP subdivisions [0, 0.0001] – very rare : (0.0001, 0.00025] (0.00025, 0.0005] (0.0005, 0.00075] (0.00075, 0.001] - rare : (0.001, 0.0025] (0.0025, 0.005] (0.005, 0.0075] (0.0075, 0.01] – low : (0.01, 0.05] – common : (0.05, 0.1] and High : (0.1, 0.5] allele frequencies.
To clarify the results in sd, each bin is represented by one unit.

72. Purcell, S. *et al*. PLINK: a tool set for whole-genome association and population-based linkage analyses. American Journal of Human Genetics 81, 559-575, doi:10.1086/519795 (2007).

73. Purcell, S. PLINK: Whole genome data analysis toolset (v 1.07). *http://pngu.mgh.harvard.edu/~purcell/plink/*. (2015).
